# Cannabinoid signalling in glioma cells

**DOI:** 10.1186/2193-1801-4-S1-L11

**Published:** 2015-06-12

**Authors:** Bozena Kaminska, Aleksandra Ellert-Miklaszewska

**Affiliations:** Nencki Institute of Experimental Biology, Warsaw, Poland

**Keywords:** Cannabinoids, signal transduction, cell death

Cannabinoids, originally derived from Cannabis sativa, as well as their endogenous and synthetic counterparts, were shown to induce apoptosis of glioma cells in vitro and tumour regression in vivo via their specific receptors, cannabinoid receptors CB1 and/or CB2. CB2 are abnormally expressed in human gliomas and glioma cell lines. Most of the analysed gliomas expressed significant levels of CB2 receptor and the extent of CB2 expression in the tumour specimens was related to tumour malignancy. A synthetic cannabinoid, WIN 55,212-2, down-regulated the Akt and Erk signalling pathways in C6 glioma cells that resulted in reduction of phosphorylated Bad levels, mitochondrial depolarization and activation of caspase cascade leading to apoptosis. We examined whether synthetic cannabinoids with different receptor specificity: WIN55,212-2 (a non-selective CB1/CB2 agonist) and JWH133 (a CB2-selective agonist) affect survival of four human glioma cell lines and three primary human glioma cell lines. WIN-55,212-2 decreased cell viability in all examined cell lines and induced cell death. Susceptibility of the cells to JWH133 treatment correlated with the CB2 expression. Cannabinoids triggered a decrease of mitochondrial membrane potential, cleavage of caspase-9 and effector caspases. Induction of cell death by cannabinoid treatment led to the generation of a pro-apoptotic sphingolipid ceramide and disruption of signalling pathways crucial for regulation of proliferation and survival. Increased ceramide levels induced ER-stress and autophagy in drug-treated glioblastoma cells. We conclude that cannabinoids are efficient inhibitors of human glioma cells growth, once the cells express specific type of cannabinoid receptor.

